# ASO Author Reflections: Enhancing Recurrence-Free Survival Prediction in Hepatocellular Carcinoma: A Time-Updated Model Incorporating Tumor Burden and AFP Dynamics

**DOI:** 10.1245/s10434-025-17342-5

**Published:** 2025-04-17

**Authors:** Miho Akabane, Timothy M. Pawlik

**Affiliations:** Department of Surgery, The Ohio State University Wexner Medical Center and James Comprehensive Cancer Center, Columbus, OH USA

## Past

Hepatocellular carcinoma (HCC) remains a global health challenge, with postoperative recurrence exceeding 40–50% within 2 years after curative-intent hepatectomy.^1,2^ Historically, prediction models have largely focused on static preoperative factors—most notably alpha-fetoprotein (AFP) and the tumor burden score (TBS)—to estimate recurrence-free survival (RFS).^3,4^ While offering valuable baseline insights, these predictors fail to capture changes in tumor biology that occur after resection. Serum AFP has long been monitored as a biomarker for HCC, reflecting active tumor growth or residual tumor cells. Prior models have not fully leveraged longitudinal AFP data, which may help inform evolving risk of recurrence. The current study investigated real-time RFS prediction by integrating time-updated AFP and tumor burden metrics.

## Present

A time-varying Cox model was developed that continuously updated RFS predictions at key postoperative intervals (e.g., at 6 and 12 months).^5^ By incorporating AFP as a time-dependent covariate, the trajectory of AFP levels was accounted for to better modeled each patient’s evolving risk profile. Analysis of 1911 patients demonstrated that while TBS remained a strong preoperative predictor, its effect on RFS diminished over time; of note, postoperative AFP values became increasingly important as a marker of prognosis with longer follow-up. The dynamic model outperformed a conventional, static preoperative Cox model. Specifically, at 6 months, the time-varying model’s C-index was 0.70 versus 0.59 for the static model; 12 months C-indices were 0.70 versus 0.56, respectively. These findings suggested that real-time incorporation of AFP trends were associated with a more accurate assessment of recurrence risk, potentially allowing clinicians to tailor surveillance intensity. Importantly, an online calculator based on this model was developed, enabling clinicians to input new AFP measurements at each follow-up visit for an updated RFS prediction (https://nm49jf-miho-akabane.shinyapps.io/AFPHCC/).

## Future

The dynamic nature of time-varying Cox models may offer improved opportunities to refine estimates of recurrence and prognosis. Rather than assessing tumor-specific factors in a static fashion, the varying nature of tumor markers such as AFP need to be considered. In addition, integrating other emerging biomarkers (e.g., protein induced by vitamin K absence-II [PIVKA-II]) or genomic/proteomic data may further enhance model predictive power. Prospective validation studies are warranted to confirm the utility of dynamic prediction models across diverse institutions and patient populations. Leveraging these types of models may help customize postoperative surveillance intervals—potentially reducing unnecessary imaging while ensuring timely detection of recurrence—leading to more personalized, cost-effective care.
